# Waterbird Species Are Highly Sensitive to Wetland Traits: Simulation-Based Conservation Strategies for the Birds of the Sicilian Wetlands (Italy)

**DOI:** 10.3390/biology13040242

**Published:** 2024-04-06

**Authors:** Alessandro Ferrarini, Claudio Celada, Marco Gustin

**Affiliations:** Lipu-BirdLife Italy, Via Pasubio 3, I-43122 Parma, Italy; claudio.celada@lipu.it (C.C.); marco.gustin@lipu.it (M.G.)

**Keywords:** avian diversity, bird conservation, conservation planning, Mediterranean bird flyway, Natura 2000 sites, wetland management

## Abstract

**Simple Summary:**

The Sicilian wetlands (Italy) witness the migration of millions of birds every year. The anthropic exploitation of these wetlands, along with the exacerbation of climate variables, could soon prevent the occurrence of many waterbird species, especially in summer, and is already impacting their abundance. Our study delivers scientifically supported planning strategies to assist with preserving and restoring the avian diversity of the Sicilian wetlands and provide wetland managers with an effective methodological framework to step down their regional-scale approach to allow for the place-based planning of existing and project wetlands.

**Abstract:**

In this study, we (a) formulated a general hypothesis about how wetland (functional and structural) traits influence avian diversity, (b) turned this hypothesis into a non-parametric Bayesian network, (c) disentangled the direct and indirect effects of the variables influencing waterbird species, and (d) simulated the changes expected to the levels of avian diversity as a result of numerous counterfactual and management scenarios. We applied our framework to the Sicilian wetlands as a whole; then, we downscaled simulations locally to a wetland of particular interest (Pantano Bruno). We found that (1) waterbird species are highly sensitive to wetland traits; (2) wetland traits have both direct and indirect effects upon alpha avian diversity; (3) the direct and indirect effects of wetland traits can be contrasting; (4) water level fluctuations (benefit), diversions (cost), and salinity (cost) are key factors for waterbird conservation; (5) these wetlands have the potential for hosting a level of alpha avian diversity that is double the baseline (from 19 to 38 species); (6) these wetlands are prone to ecological collapse if all traits deteriorate (from 19 to 6 species per wetland); and (7) the ecological information gained at the regional scale can be properly downscaled to the local scale to make inferences on single wetlands.

## 1. Introduction

Healthy and well-functioning wetlands supply a variety of ecosystem functions and services, including carbon fixation, increased flood prevention, improved water quality, pollutant filtration services, and soil melioration [1,2]. Wetland benefits are not exclusively ecological; in fact, climate regulation, the support of productive fisheries, and recreational opportunities have important economic impacts as well [3]. Wetlands also supply habitats, food, and shelter for waterbirds [4,5] and act as stopover sites that allow waterbirds to migrate [6]. Despite their exceptional ecological and economic value [7,8], more than half of the world’s wetlands have been altered, degraded, or lost in the last 150 years [2,3]. Tourism, agricultural expansion, and urban development are some of the major causes of this decline [9,10]. In addition, climate change, with warmer and dryer summer periods, could soon worsen the risk of the complete drainage of wetlands in summer [1,11].

As a result of their position at the land–sea interface, coastal wetlands are particularly vulnerable to climate change [12,13]. Besides the common threats acting upon wetland ecosystems, climate-induced sea level rises will increase the frequency of saltwater intrusions in coastal wetlands [14]. The increased evaporation caused by climate warming, combined with saltwater intrusions, could alter these ecosystems and decrease their species richness [15].

The coastal wetlands of Sicily (Italy) belong to the central–eastern Mediterranean bird flyway, i.e., a migration route of high importance for a large number of avian species crossing the Mediterranean Sea, and behave as refueling areas for hundreds of thousands of waterbirds that make long migratory journeys between Africa and Europe [16,17]. The Sicilian wetlands are thus rated among the most interesting natural sites at the European level [18]. In summer (July–September), these wetlands host > 70 waterbird species (Table S1), of which almost 90% are migratory; thus, their conservation interest is primarily at the European and global levels [19]. The pied avocet, the little bittern, the black-winged stilt, and the ferruginous duck are of major conservation interest in these wetlands [20]. Despite their ecological importance, the coastal wetlands of Sicily are severely impacted by human activities and ongoing climate change. In 1990–2012, a constant increase in agricultural areas and a decrease in natural and semi-natural ones occurred in the close surroundings of these wetlands [21]. Water diversion for agricultural activities, the anthropization and degradation of the surroundings, and tourism pressure are nowadays common threats to these wetland and waterbird species [20]. In addition, climate change has already increased the frequency of complete wetland drainage in summer (due to increased evaporation) and saltwater intrusions (due to accelerated sea level rises) [19]. Accordingly, the provision of suitable habitats for waterbirds has become a main goal of wetland management and restoration in Sicily.

In this study, we moved beyond the identification of the status of, and threats to, these coastal wetlands, and built a simulation framework, fed with field data, to address questions about how avian diversity in the Sicilian wetlands relates to wetland (functional and structural) traits. Formally, our objectives in this study were to (a) understand the degree to which alpha avian diversity (i.e., the mean number of avian species per wetland) is a function of spatial, anthropic, and hydrological wetland traits and (b) provide insights into how different management and restoration strategies applied to such traits could regulate levels of alpha avian diversity. Firstly, we applied our framework to the coastal wetlands of Sicily as a whole; secondly, we downscaled our simulations locally to the Pantano Bruno, an unmanaged wetland of particular interest for its potential to host waterbird species and because it is located near the wetlands of Cuba and Longarini that are successfully managed by the German foundation Stiftung Pro Artenvielfalt.

## 2. Materials and Methods

### 2.1. Site Selection

**Figure 1:**
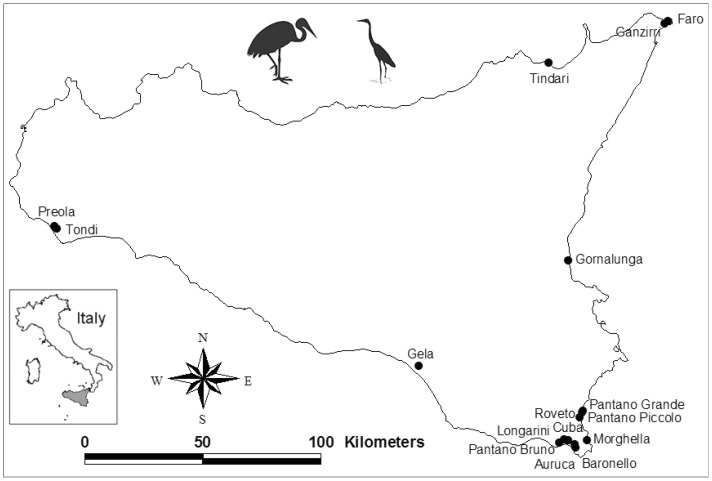
Study area (Sicily, Italy). The sixteen coastal wetlands (total surface area = 676 hectares) under study are shown. With the exceptions of Faro and Ganzirri, all wetlands belong to the Natura 2000 network.

We selected all natural coastal wetlands larger than 10 hectares, also including the temporary wetlands that are common in summer as a result of evaporation, decreases in rainfall, and water diversions for agricultural activities [19]. We excluded artificial wetlands (e.g., fish and shrimp ponds, farm ponds, salt flats, etc.). As a result of this selection procedure, we chose 16 natural coastal wetlands (Figure 1), of which 14 belong to the Natura 2000 network [22]. In Sicily, almost all inland wetlands are artificial, and the few natural wetlands that there are did not meet our minimum wetland size criterion; hence, the coastal wetlands selected in this study represent almost entirely the system of natural wetlands present in this region.

The wetland Pantano Bruno (coordinates: 36°41′56.72″ N, 14°58′55.58″ E) on the south-east coast of Sicily belongs to the Natura 2000 network, both as a special protection area (SPA ITA090029; Birds Directive) and a special area of conservation (SAC ITA090003; Habitats Directive). It is part of a broad subsidence plain where slow ground uplift and eustatic sea level fluctuations created a long line of dunes that separate the inner part of this plain from the open sea, after which the former basin was flooded and formed this wetland (along with the nearby wetlands Cuba and Longarini). Thanks to the fertile soil around, farming activity (vegetable growing, grain crops, and viniculture) has increasingly intensified in the area in recent decades, and numerous polytunnel greenhouses are now immediately adjacent to this wetland. As a result, water discharges and diversions from greenhouses are rather frequent in summer as well.

### 2.2. Field Surveys

For each wetland, we used regularly spaced sampling points (the number of sampling points was proportional to the wetland extent, with a maximum of 12 points with a 200 m minimum distance to minimize spatial autocorrelation [23]) where we applied the standard point count sampling method (i.e., a 100 m observation distance around each sampling point and a 15 min observation time with recording of all visual contacts [24]). We collected 58 sampling points (i.e., a mean of 3.6 sampling points per wetland) where we performed five sampling sessions of avian diversity at regular intervals of 10–15 days during July–September 2016. We also assigned three spatial (wetland size, isolation, and distance to the coastline), two anthropic (tourism pressure and anthropization of the surroundings), and five hydrological (mean water level, water level fluctuations, water salinity, discharges, and diversions) traits to each wetland (Table S2). We measured five wetland traits on a semi-quantitative scale of 0 to 3 using both the authors’ and local (the natives, local policymakers, and landowners) expertise. Each expert supplied an independent score for each threat (0 = absent, 1 = localized, 2 = scattered, 3 = widespread). In the few cases of disagreement, on a precautionary basis, we chose the most elevated score among those provided by the experts. The ten wetland traits are detailed in Table S3. Biodiversity sampling was carried out before the assessment of wetland traits to avoid the disturbance of avifauna and thus potential biases in the sampling process.

### 2.3. Model Calibration

**Figure 2:**
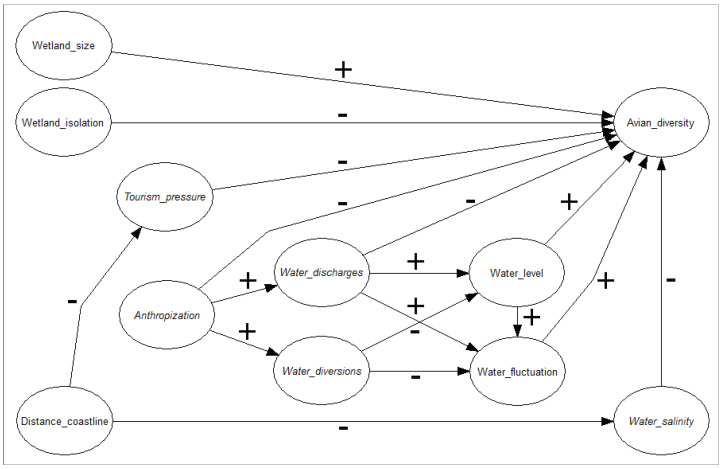
Metamodel representing our general hypothesis of the key interrelationships that regulate the levels of avian diversity in the coastal wetlands of Sicily as a function of spatial, anthropic, and hydrological variables. Arrows denote the hypothesized direct effects among variables. The symbols “+” and “−” indicate positive and negative effects, respectively.

We first elaborated upon a conceptual metamodel based on hypothesized pathways of key dependencies that could determine alpha avian diversity in the wetlands under study (Figure 2). We based the metamodel on our fieldwork experience [19,20,21].

Second, we developed a non-parametric Bayesian network (NBN; [25,26,27]), fed with the measured data at our field locations, that assimilated the hypothesized pathways of the metamodel into a network of all the distal and proximal variables that were expected to rule the alpha avian diversity in the Sicilian wetlands. An NBN model can deal with both discrete (defined in an ordinal scale) and continuous distributions, which is essential when dealing with (proxy) ordinal variables, as in our case study. Once the model structure was conceptualized (Figure 2), the marginal distributions of the variables and the partial correlations between them were calculated using the empirical data from our field surveys.

The nodes represented univariate random variables (*x_1_*, *x_2_*, …, *x_n_*) and the partial correlations were calculated using the normal copula [28]. Any joint cumulative distribution function (*F_1_*…*F_n_*) of variables *x_1_*…*x_n_* could be rewritten in terms of the corresponding copula *C* as

*F_1_*_…*n*_ (*x_1_*…*x_n_*) = *C*(*F_1_*(*x_1_*)…*F_n_*(*x_n_*))

where *F_i_*(*x_i_*) is the marginal distribution of the *i*-th variable. Since this study involved discrete (ordinal) and continuous data, the Gaussian (normal) copula was selected here as the most proper technique [29], and calculated as

*F_1…n_ (x_1_…x_n_) = C_ρ_(F_1_(x_1_)…F_n_(x_n_); ρ)*

where *C_ρ_* is the joint Gaussian copula function and *ρ* denotes the partial correlations between variables. In order to build the NBN model, we employed the UninetEngine package [25].

### 2.4. Disentangled Causal Effects on Avian Diversity

After the model was calibrated, we disentangled the direct and indirect effects of each wetland trait upon avian diversity. The direct effect (*E_D_*) of the generic variable *x_i_* was simply the partial correlation (*ρ_iAD_*) between *x_i_* and alpha avian diversity (i.e., the mean number of waterbird species per wetland; *AD*). Instead, the indirect effect (*E_I_*) was calculated as

$$E_{I}=\sum_{k=1}^{n}\left(\prod_{{P_{k}}^{x_{i}\to AD}}\rho_{k}\right)$$

where *P_k_^xi^^→^^AD^* is the generic *k*-th pathway connecting the generic variable *x_i_* to *AD* through several intermediate variables and *ρ_k_* symbolizes the partial correlations present along *P_k_^i^^→^^AD^*. The direct pathway between *x_i_* and *AD* was excluded from the computation of *E_I_* because it represented here the direct effect *E_D_*.

Finally, the total effect (*E_T_*) of each wetland trait on *AD* was computed as

$$E_{T}=E_{D}+E_{I}=\rho_{iAD}+\sum_{k=1}^{n}\left(\prod_{{P_{k}}^{x_{i}\to AD}}\rho_{k}\right)$$

A plain exemplification of how *E_T_* was calculated is provided in Figure S1.

### 2.5. Model Validation

As per [26], we tested whether joint Gaussian copulas adequately represented the measured data at our field locations. Two determinants were computed [27]: *D_E_* (determinant of the empirical rank correlation matrix, i.e., the dependence structure of the original data) and *D_N_* (determinant of the empirical normal rank correlation matrix, i.e., the dependence structure of the normal copula assumption). The empirical rank correlation matrix was calculated by using Spearman’s *rho* correlation coefficient:

$$rho=1-(6*\sum_{i=1}^{n}{d_{i}}^{2}/(n^{3}-n))$$

where *n* is the number of wetlands and *d_i_* is the rank of the *i*-th wetland in the first variable minus the rank of the *i*-th wetland in the second variable.

The two determinants *D_E_* and *D_N_* were calculated as

$$D=\prod_{i,j}(1-{\rho^{2}}_{ij})$$

where *ρ_ij_* is the partial correlation between the generic variables *i* and *j*. *D* reached 1 if all variables were independent and 0 in the case of multivariate linear dependence.

By resampling the joint normal distribution 10^4^ times, we achieved the distribution of *D_N_* and extracted the 5-th and 95-th quantiles of this distribution. We then checked whether *D_E_* was within these bounds (i.e., 90% confidence interval of *D_N_*); if so, the normal copula assumption could not be rejected at the 10% significance level [26].

### 2.6. Simulations

After the NBN was calibrated and validated successfully, we performed several what-if simulations by conditionalization, i.e., by setting the value of one or more variables of the NBN to infer how it/they affected the state of the target variable (*AD*). Changes to each variable were propagated through the Bayesian network, causing direct and indirect effects on all other variables. The strength of such effects depended on how much the variables influenced each other (i.e., partial correlations *ρ_ij_*).

The baseline scenario (S_0_) represented the level of alpha avian diversity in 2016 (i.e., no conditionalization), against which the other scenarios could be compared. It should be noted that the environmental traits of these wetlands in 2016 had already deteriorated because of human impacts; thus, the baseline scenario does not represent here an ideal condition for comparison but just a temporal benchmark for future scenarios. We performed twenty what-if simulations (S_1_…S_20_), of which the first seven (S_1_…S_7_) simulated the effects on alpha avian diversity when no conservation and restoration measures were taken (worst-case counterfactual scenarios). To this aim, we simulated the generalized increase in (a) tourism pressure, (b) the anthropization of the close surroundings, (c) water salinity, and (d) water diversions and the generalized decrease in (e) water discharges and (f) water level fluctuations. Unlike the Sardinian wetlands [30,31], in summer, many Sicilian wetlands have low water levels (<15 cm; Pantano Cuba, Pantano Longarini, Pantano Morghella, Pantano Bruno, Pantano Grande, and Pantano Roveto; Table S2); thus, water discharges, although rich in pollutants, have an overall positive effect on many bird species as they represent the only alternative to complete drainage [20]. This is why the decrease in water discharges was considered among the worst-case scenarios. In the management scenarios (S_8_…S_14_), some conservation and/or restoration measures counteracted the expected trends of the variables influencing avian diversity. In the mixed scenarios (S_15_…S_20_), all the conditions deteriorated except for one, which was counteracted by some type of conservation and/or restoration measure. We then downscaled the NBN locally to the wetland Pantano Bruno and simulated a further 30 scenarios belonging to the worst-case (S_21_…S_33_), best-case (S_34_…S_45_), and mixed (S_46_…S_50_) categories.

## 3. Results

### 3.1. Sicilian Wetlands

**Figure 3:**
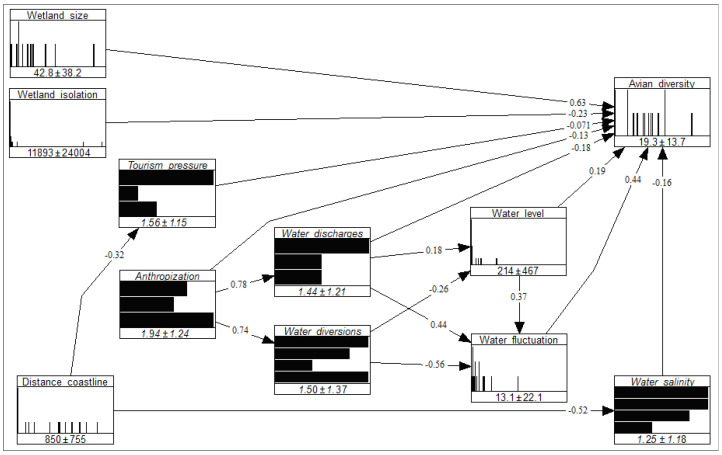
Non-parametric Bayesian network representing connections between alpha avian diversity and wetland traits in the coastal wetlands of Sicily in summer. For each variable, numbers indicate the mean and standard deviation. Values on the arcs are partial correlation coefficients between variables.

Figure 3 depicts the NBN results. The model validation was successful (i.e., the partial correlation matrix under the normal copula assumption provided an adequate approximation of the partial correlation matrix of the field data). In fact, *D_E_* was 2.59 × 10^−5^ and fell between the 0.75 and 0.80 quantiles of the confidence band of *D_N_* (the 5-th and 95-th quantiles equal to 1.38 × 10^−5^ and 6.15 × 10^−4^, respectively; Figure S2).

The disentanglement of the direct and indirect effects of the wetland traits on alpha avian diversity (Table 1) showed that (a) five traits only had direct effects; (b) two traits only had indirect effects; (c) three traits had both direct and indirect effects; (d) water discharges had opposite direct (negative) and indirect (positive) effects, with the indirect effect prevailing over the direct one; (e) the largest positive effect (due to wetland size) was only direct; (f) the largest negative effect (due to water diversions) was only indirect; (g) the distance to the coastline had a positive (indirect) effect due to its negative influence on two traits (tourism pressure and water salinity) that negatively affected alpha avian diversity; (h) the direct (negative) effect of anthropization was largely prevalent, more than the indirect one.

The baseline level of the alpha avian diversity (*AD*) of the Sicilian wetlands was 19.3 species (±13.7 S.D.). The worst-case scenarios (Table 2) showed the elevated impact of water level fluctuations, diversions, and salinity on *AD*. All other wetland traits being equal, in case the water level fluctuations became null in all wetlands (scenario S_6_), *AD* was expected to decrease by 9 species (from 19.3 to 10.3). Water diversions were the second most important type of impact on waterbirds. *Ceteris paribus*, if water diversions became widespread in all wetlands (scenario S_5_), *AD* was expected to decrease by nearly 4 species (from 19.3 to 15.5). Water salinity was the third most important type of impact on the avifauna. With all other traits unchanged, if salinity became widespread in all wetlands (scenario S_2_), *AD* was expected to decrease by 3 species (from 19.3 to 16.2). *AD* was expected to decline by 68.2% (from 19.3 to 6.14 species) in the worst possible scenario (S_7_).

The best-case scenarios (Table 2) indicated that the wetland traits that most threatened the waterbirds of the Sicilian wetlands could also serve as a trigger for their conservation if properly managed. All else being equal, if the water level fluctuations of the studied wetlands were equal to 20 cm, then *AD* could increase up to 26.7 (scenario S_13_). This positive effect was expected to hold up to 30 cm, after which, further increments in water level fluctuations would not produce further increments in *AD* (Figure S3). All other variables being constant, the prohibition of water diversions (scenario S_12_) in the 10 wetlands where they occur (Table S2) was expected to determine an increase in *AD* of almost four species (from 19.3 to 23.2). All else being the same, the desalination of the brackish water in the 10 wetlands where seawater intrusions were present (Table S2) was predicted to enhance *AD* by 8.5% (from 19.3 to 21.5 species; scenario S_9_). *AD* was expected to equal 38.5 (a 99.4% increment) in the best possible scenario (S_14_).

The mixed scenarios (Table 2) showed that one single conservation measure could not preserve the baseline levels of avian diversity if all other wetland traits deteriorated.

A map showing the geographical pattern of the alpha avian diversity in summer in the coastal wetlands of Sicily is shown in Figure S4.

### 3.2. Wetland Pantano Bruno

After setting the values of all NBN variables to those of the Pantano Bruno wetland (i.e., Bayesian conditionalization), the predicted *AD* was 19 (Figure S5), which corresponded exactly to the level of avian diversity sampled in this wetland (Table S2). All other wetland traits being constant, an increase in water diversions (from “*scattered*” to “*widespread*”) was expected to decrease *AD* to only 12 species (scenario S_24_; Table 3). All else being the same, a slight decrease in water level fluctuations (from 6.8 to 5 cm) would be enough to produce a drop of 3 species (from 19 to 16; scenario S_25_); in case water level fluctuations decreased to 0 cm, *AD* was predicted to decline by 57.9% (from 19 to 8 species; scenario S_26_). *Ceteris paribus*, the concomitant increase in water salinity (from “*absent*” to “*widespread*”) and water diversions (from “*scattered*” to “*widespread*”) would lead to a 52.6% decrease in *AD* (from 19 to 9 species; scenario S_29_). The worst possible scenario (S_33_) depicted an ecological bottleneck, with only 3 avian species present in this wetland.

The best-case scenarios (S_34_…S_45_; Table 3) showed great potential for waterbird conservation, provided by the control of water level fluctuations. All else being equal, if this wetland trait increased to 15 cm (scenario S_38_) and 30 cm (scenario S_41_) from the current level (6.8 cm), *AD* was predicted to grow from 19 to 25 and 29 species, respectively. The best possible scenario (S_45_) predicted an increase in *AD* of 15 species (78.9% increase).

The mixed scenarios for the wetland Pantano Bruno (S_46_…S_50_; Table 3) suggested the potential for preserving the baseline levels of avian diversity, even in the presence of adverse wetland traits. For example, water level fluctuations equaling 15 cm could compensate for an increase (from “*absent*” to “*widespread*”) in water salinity (scenario S_50_).

## 4. Discussion

Many recent studies have clearly evidenced the unfavorable effects of human [32] and climate [33] threats on wetlands and waterbirds. Researchers are thus impelled to employ new approaches to the targeted management of these ecosystems and the associated biodiversity. Because wetland management and planning are arguable issues with potential for conflict [34], a methodological framework capable of testing many different scenarios and exploring their implications for biodiversity conservation is highly advisable. Simulation modeling, combined with field surveys, effectively addressed this matter. Modeling and simulating changes to the levels of avian diversity as a function of different plausible scenarios provided a sound approach to dealing with intrinsic uncertainties in wetland and waterbird conservation planning.

We found that (a) the waterbird species of the study area are highly sensitive to wetland traits; (b) wetland traits have both direct and indirect effects upon alpha avian diversity; (c) the direct and indirect effects of wetland traits can be contrasting; (d) water level fluctuations (the higher the better), diversions (the lower the better), and salinity (the lower the better) are key factors for waterbird conservation in the study area; (e) water discharges have an overall positive effect on many bird species as they counteract the excessive decrease in water levels due to water diversions, evaporation, and lack of rainfall in the summer period; (f) the coastal wetlands of Sicily have potential for hosting a level of alpha avian diversity that is double the baseline (from 19 to 38 species per wetland in the best possible scenario); (g) at the same time, these wetlands are prone to ecological collapse if all traits deteriorate (from 19 to 6 species per wetland in the worst possible scenario); (h) the difference in the levels of alpha avian diversity between the best and worst possible scenarios is huge (32 species); (i) the ecological information gained at the regional scale can be properly downscaled to the local scale to make inferences on single wetlands; (j) the wetland Pantano Bruno can be either a hotspot (34 species in the best possible scenario) or an unimportant area (3 species in the worst possible scenario) for avian diversity; (k) the mixed scenarios for this wetland can effectively preserve the baseline levels of avian diversity, even in the presence of adverse wetland traits.

### 4.1. Methodological Issues

Because field data were collected during July–September, the results of this study hold only for the summer period and cannot in any way be extrapolated to the other periods of the year.

We focused on the summer period because it is a bottleneck time for waterbirds due to higher levels of tourism activities and anthropization (illegal dumpsites, camping sites, caravan parks, and so forth) and the concomitant decrease in water levels ascribable to water diversions, evaporation, and low rainfall [19,20,21]. In addition, because the Sicilian wetlands belong to the central–eastern Mediterranean bird flyway, in summer, it hosts the highest number of individual birds and species during migration [5,16]. Thus, the proper management of these wetlands is particularly challenging during this critical period of the year and demands scientifically supported planning strategies to assist with preserving, or restoring, the levels of avian diversity.

We did not include hunting pressure among the wetland traits because there is a complete hunting ban in the studied sites; in fact, all the wetlands included were in either natural reserves or oriented natural reserves. However, we cannot exclude episodes of bird poaching in these areas.

Our simulation framework did not take into account the role of species co-occurrences in determining the levels of avian diversity. In the study area, waterbird species individualistically colonize the wetlands where they encounter appropriate conditions [20]. Under these circumstances, species act independently of one another, species associations are weak, and environmental control is largely prevalent [35].

Further methodological details are discussed in Table S4.

### 4.2. Management Implications

Europe conserves biodiversity through the “Natura 2000” ecological network of protected areas, the largest such network in the world [36]. The management plan, conservation measures, and appropriate assessments (art. 6 of EU Habitat Directive) are the basis for the conservation of species and habitats in the Natura 2000 sites [22]. Because the Sicilian wetlands almost completely belong to the Natura 2000 network and witness the migration of millions of birds every year, the elevated difference in the levels of avian diversity between the best and worst possible scenarios demands that local administrations better preserve and restore these wetlands and waterbirds by updating their governing plans. In addition, these wetlands also belong to the network of natural regional reserves of Sicily; as such, the regional management plans for this network should be promptly updated as well.

Water level fluctuations largely regulate the levels of alpha avian diversity in the Sicilian wetlands. Water level variations create habitats with different water depths, thus providing more foraging opportunities for birds [2,4]. Increasing water level fluctuations during the summer period is thus of paramount importance to preserve, or increase, the levels of alpha avian diversity. We found that water level fluctuations between 20 and 30 cm would be ideal in the study area (Figure S3), but 10 out of 16 wetlands had values < 10 cm (Table S2). The wetland Pantano Bruno would benefit from an increase in water level fluctuations of at least 8 cm (from 6.8 to 15 cm; Table 3), which roughly corresponds to discharge and the removal at regular intervals of 13,200 m^3^ of water (i.e., wetland size × change in water depth = 165,000 m^2^ × 0.08 m). In Sicily, on average, 4673 m^3^ of water is used yearly for each irrigated hectare [21]; thus, this value corresponds to the volume of water used to irrigate 2.82 hectares of agricultural areas yearly. To some extent, water discharges in many Sicilian wetlands already determine some increases in water levels and water level fluctuations; in fact, they resulted in an overall positive (although weak) effect on alpha avian diversity. However, even if short-term effects of water pollution discharges from the surrounding agricultural areas (mostly greenhouses) are positive, their mid- and long-term consequences on waterbirds are unknown, and could in all probability cause illnesses and a decrease in the reproductive success and fitness of birds [37]. By contrast, water diversions depressed water level fluctuations in many wetlands and often forced water levels to approach zero (Table S2), thus hindering the natural process of water level fluctuations due to summer evaporation. Consequently, two alternative solutions are necessary to increase water level fluctuations: (a) the prohibition of water diversions for agricultural activities before and during the summer period and (b) the use of water delivery and water discharge structures (e.g., hydraulic pumps) to artificially raise and lower water levels. The former management strategy is urgent in five wetlands (Pantano Auruca, Pantano Bruno, Pantano Cuba, Pantano Longarini, Pantano Morghella; Table S2) and the latter in four (Pantano Baronello, Pantano Grande, Pantano Piccolo, Pantano Roveto; Table S2). Once these policies have been applied, water discharges would no longer be useful to sustain avian diversity in the study area; on the contrary, they could be prohibited in order to reduce their direct (and negative) effect on avian diversity in at least six wetlands (Pantano Auruca, Pantano Baronello, Pantano Bruno, Pantano Longarini, Pantano Morghella, Pantano Roveto; Table S2).

Water salinity negatively influenced the levels of avian diversity in the Sicilian wetlands. Many waterbird species avoid saline waters as they cause (a) weight loss by dehydration, (b) increases in the energy costs of thermoregulation, and (c) reductions in the waterproofing of feathers [38]. Water salinity also affects the species composition of the aquatic plant communities and vegetation of the wetland shoreline, which impacts herbivorous waterbirds [4]. Seven wetlands are already affected by scattered (Lago Faro, Lago Ganzirri, Lago Gornalunga, and Pantano Grande) or widespread (Pantano Longarini, Pantano Roveto, and Laghetti Tindari) saltwater intrusions (Table S2), while the wetland Pantano Bruno is not yet impacted. Furthermore, sea levels are expected to increase by almost 9 cm by 2040 [19], which will further increase the frequency of saltwater intrusions and even the risk of seaward wetland losses for four wetlands adjoining the coastline (Lago Gornalunga, Pantano Morghella, Pantano Piccolo, Pantano Roveto; Table S2). The changing inundation and salinity regimes compel local administrations and stakeholders to plan (a) the closure of all channels that connect these wetlands to the sea and (b) the construction of coastal defense structures around these seven wetlands (for example, in the form of artificial dune cordons at least 10 cm high). Another cost-effective solution to the problem of water salinity could be the cultivation of highly tolerant wild plant species for phytoremediation purposes. Plants with high biomass, adapted to local climate conditions, and with elevated phytoextraction (absorption and accumulation in their tissues) of salt from wetland water are ideal candidates for this purpose [39].

The anthropization of the surroundings of the Sicilian wetlands is mostly due to the construction of greenhouses for fruit-growing (oranges, tangerines, grapes, and lemons; [21]). Besides the increase in water discharges and diversions, the anthropization of the surroundings subtracts natural space from the wetland shoreline that could act as a resting and foraging habitat for waterbirds. Coastal wetlands can adapt to rising seas by moving landward into adjacent upslope or upriver ecosystems [40]. The potential for wetland adaption to sea level rises is governed in part by the availability of accommodation space (i.e., the vertical and lateral space available for wetland establishment in response to rising seas). Because the landward movement of wetlands comes at the expense of adjacent lands, the anthropization of the surroundings is an impediment to wetland adaptation to sea level rises. The construction of new greenhouses should thus be prohibited everywhere in the close surroundings of the Sicilian wetlands, especially for wetlands adjacent to the coastline (Lago Gornalunga, Pantano Morghella, Pantano Piccolo, Pantano Roveto; Table S2). The removal of some illegal dumpsites present in the surroundings of some wetlands [19] is also necessary.

Tourist and recreational activities have manifold effects on waterbirds, including noise disturbance, damage to the habitat, and temporary occupancy of the space around the wetlands, which can lead to reduced breeding success and modified habitat use [2]. These activities should be prohibited around the wetlands that host the highest levels of avian diversity (Pantano Roveto, Biviere Gela, Lago Gornalunga, and Pantano Baronello; Table S2) and at least limited around those wetlands where they are already widespread (Lago Faro, Lago Ganzirri, Pantano Longarini, Pantano Grande, and Laghetti Tindari; Table S2).

In addition, the massive size reduction of the Sicilian wetlands in the last century and the anthropization of the surroundings in the past 30 years [21] urge the creation of new coastal wetlands, which would support the objectives of the Birds Directive and the EU 2030 Biodiversity Strategy. In this view, our methodological approach provides a further and valuable opportunity for wetland planners. The creation of new wetlands requires considering a large number of site-specific traits [37]; therefore, site selection is an essential step for planning new wetlands. The characteristics of existing wetlands in the same area, or in areas with similar characteristics, can be used as models for what might be expected of the project wetland. For example, researchers can build a non-parametric Bayesian network model linking wetland traits to avian diversity in a certain area and then simulate the expected levels of avian diversity of a project wetland as a function of many different combinations of site-specific (e.g., isolation, distance to the coastline), structural (e.g., size), and functional (e.g., hydrological, anthropic) traits. In essence, this is exactly what we have realized for the wetland Pantano Bruno in this study, with the difference that the conditioning of the proposed model would be on a wide range of simulated values rather than on the actual trait values of a specific wetland. This kind of application of non-parametric Bayesian modeling would provide wetland managers the opportunity to step down their regional-scale planning to allow for the place-based planning of new wetlands as well.

Our methodological approach can also replace experimental approaches to evaluating the effects of wetland management on waterbird species richness and abundance. For example, Taft et al. [41] examined the effect of drawdown on waterbird communities by using controlled experiments that compared replicated treatments of “drawdown” and “no drawdown”. For each experiment, they tested the hypothesis that waterbird species richness and abundance increased or decreased over time in response to such treatments. However, experimental approaches to evaluating wetland management are rare in wildlife research because they are costly, and rigorous experimentation with sufficient statistical power is difficult at large spatial scales [42,43]. In this view, the in silico simulation modeling provided here a solution to address this matter effectively and inexpensively as it allowed for the testing of numerous management scenarios prior to field interventions.

## 5. Conclusions

Our methodological approach provided a robust framework to (a) formulate clear hypotheses on how relevant variables regulate the avian diversity in the coastal wetlands of Sicily, (b) validate this hypothesis by comparing correlation structures of field data with correlation structures of the proposed model, and (c) simulate changes to wetland traits and predict their effects on avian diversity.

We found that many of the processes connecting wetland traits and avian diversity described in the scientific literature operate simultaneously as parts of a whole system of cascading effects. Accordingly, our approach supplies planners with a systems-level understanding of wetland traits–avian diversity relationships, improves chances to foresee the consequences of the human alteration of wetlands, and helps discover how we could mitigate and/or compensate such consequences upon biodiversity.

Further studies on these wetlands could (a) extend the study period both year by year and by lengthening the fieldwork seasons to the winter period when the Sicilian wetlands accumulate a large number of wintering aquatic birds from most of Europe and (b) assess the potential impact of poaching activities on bird abundance and alpha diversity.

In light of our results, we established consistency in the management and restoration of these wetlands by providing an informed and scientifically defensible basis for proactive and targeted planning strategies. 

## Figures and Tables

**Table 1:** Direct, indirect, and total effects of wetland traits upon the alpha avian diversity in the coastal wetlands of Sicily. The total effect was simply the sum of the direct and indirect effects.

Wetland Trait	Direct Effect on Alpha Avian Diversity	Indirect Effect on Alpha Avian Diversity	Total Effect on Alpha Avian Diversity
Anthropization	−0.13	−0.02	−0.15
Distance to the coastline	0.00	0.11	0.11
Mean water level	0.19	0.16	0.35
Tourism pressure	−0.07	0.00	−0.07
Water discharges	−0.18	0.23	0.05
Water diversions	0.00	−0.30	−0.30
Water level fluctuations	0.44	0.00	0.44
Water salinity	−0.16	0.00	−0.16
Wetland isolation	−0.23	0.00	−0.23
Wetland size	0.63	0.00	0.63

**Table 2:** Expected impacts of twenty what-if scenarios upon the levels of avian diversity (last column) in the coastal wetlands of Sicily in summer. The scenario S_0_ (baseline scenario) represented the distribution of avian diversity in 2016 and was here the reference point against which the other scenarios could be compared. Water discharges benefitted avian diversity for the reasons explained in the main text.

Code	Counterfactuality	Conditionalization	Avian Diversity (Mean ± S.D.)
S_0_	none	none	19.3 ± 13.7
S_1_	tourism pressure is widespread in all wetlands	tourism pressure = 3	18.2 ± 12.8
S_2_	water salinity is widespread in all wetlands	water salinity = 3	16.2 ± 12.1
S_3_	water discharges are null in all wetlands	water discharges = 0	17.7 ± 12.8
S_4_	anthropization is widespread in all wetlands	anthropization = 3	17.9 ± 12.6
S_5_	water diversions are widespread in all wetlands	water diversions = 3	15.5 ± 11.7
S_6_	water level fluctuations are null in all wetlands	water level fluctuations = 0 cm	10.3 ± 8.7
S_7_	all conditions deteriorate (worst possible scenario)	scenarios from S_1_ to S_6_ together	6.1 ± 6.6
S_8_	tourism pressure is null in all wetlands	tourism pressure = 0	19.8 ± 13.3
S_9_	water salinity is null in all wetlands	water salinity = 0	21.5 ± 13.6
S_10_	water discharges are widespread in all wetlands	water discharges = 3	20.4 ± 13.4
S_11_	anthropization is null in all wetlands	anthropization = 0	20.3 ± 13.4
S_12_	water diversions are null in all wetlands	water diversion = 0	23.2 ± 13.9
S_13_	water level fluctuations are 30 cm in all wetlands	water level fluctuations = 30 cm	29.7 ± 14.0
S_14_	all conditions improve (best possible scenario)	scenarios from S_8_ to S_13_ together	38.5 ± 13.6
S_15_	all conditions deteriorate but tourism pressure is null	same as S_7_ but tourism pressure = 0	6.6 ± 6.8
S_16_	all conditions deteriorate but water salinity is null	same as S_7_ but water salinity = 0	8.4 ± 8.2
S_17_	all conditions deteriorate but water discharges are widespread	same as S_7_ but water discharges = 3	8.1 ± 8.4
S_18_	all conditions deteriorate but anthropization is null	same as S_7_ but anthropization = 0	7.9 ± 6.1
S_19_	all conditions deteriorate but water diversions are null	same as S_7_ but water diversions = 0	9.7 ± 7.3
S_20_	all conditions deteriorate but water level fluctuations equal 30 cm	same as S_7_ but water level fluctuations = 30 cm	20.6 ± 11.8

**Table 3:** Expected impacts of thirty what-if scenarios upon the levels of avian diversity (last column) in the wetland Pantano Bruno in summer. The scenario S_0_ (baseline scenario) represented the number of waterbird species in 2016 and was here the reference point against which the other scenarios could be compared. The cells in grey indicate the simulated changes with respect to S_0_. The letters among parentheses beneath each wetland trait indicate whether it was a benefit (B) or a cost (C) for the waterbird species. Water discharges benefitted avian diversity for the reasons explained in the main text.

Code	Counterfactuality	Water Level Fluctuations (B)	Water Salinity (C)	Water Diversions (C)	Water Discharges (B)	Tourism Pressure (C)	No. of Waterbird Species
S_0_	none	6.8	0	2	2	1	19
S_21_	increase in water salinity	6.8	3	2	2	1	14
S_22_	increase in tourism pressure	6.8	0	2	2	3	18
S_23_	decrease in water discharges	6.8	0	2	0	1	15
S_24_	increase in water diversions	6.8	0	3	2	1	12
S_25_	water level fluctuations = 5 cm	5.0	0	2	2	1	16
S_26_	water level fluctuations = 0 cm	0.0	0	2	2	1	8
S_27_	S_21_ and S_22_ together	6.8	3	2	2	3	13
S_28_	S_21_ and S_23_ together	6.8	3	2	0	1	11
S_29_	S_21_ and S_24_ together	6.8	3	3	2	1	9
S_30_	S_21_ and S_25_ together	5.0	3	2	2	1	12
S_31_	S_21_ and S_26_ together	0.0	3	2	2	1	4
S_32_	S_21_, S_23_, and S_25_ together	5.0	3	2	0	1	10
S_33_	worst possible scenario	0.0	3	3	0	3	3
S_34_	decrease in tourism pressure	6.8	0	2	2	0	20
S_35_	increase in water discharges	6.8	0	2	3	1	21
S_36_	decrease in water diversions	6.8	0	0	2	1	24
S_37_	water level fluctuations = 10 cm	10.0	0	2	2	1	20
S_38_	water level fluctuations = 15 cm	15.0	0	2	2	1	25
S_39_	water level fluctuations = 20 cm	20.0	0	2	2	1	25
S_40_	water level fluctuations = 25 cm	25.0	0	2	2	1	25
S_41_	water level fluctuations = 30 cm	30.0	0	2	2	1	29
S_42_	S_35_ and S_37_ together	10.0	0	2	3	1	23
S_43_	S_35_ and S_41_ together	30.0	0	2	3	1	31
S_44_	S_34_ and S_37_ together	10.0	0	2	2	0	21
S_45_	best possible scenario	30.0	0	0	3	0	34
S_46_	S_21_, S_35_, and S_36_ together	6.8	3	0	3	1	17
S_47_	S_25_, S_35_, and S_36_ together	5.0	0	0	3	1	20
S_48_	S_26_, S_35_, and S_36_ together	0.0	0	0	3	1	11
S_49_	S_22_, S_24_, S_35_, and S_41_ together	30.0	0	3	3	3	22
S_50_	S_21_ and S_38_ together	15.0	3	2	2	1	19

## Data Availability

Data are available via the Researchgate Digital Repository https://www.researchgate.net/publication/378877408_dataset_waterbirds_Sicilian_wetlands, accessed on 8 January 2024.

## Supplementary Materials

The following supporting information can be downloaded at https://www.mdpi.com/article/10.3390/biology13040242/s1: Figure S1: A plain exemplification of how direct, indirect, and total effects of the generic variable *x_i_* on alpha avian diversity were calculated; Figure S2: Validation of the non-parametric Bayesian network; Figure S3: Alpha avian diversity as a function of water level fluctuations in the Sicilian wetlands in summer; Figure S4: Map showing the geographical pattern of the alpha avian diversity in summer in the coastal wetlands of Sicily; Figure S5: Conditioning of the non-parametric Bayesian network on the attributes of the wetland Pantano Bruno; Table S1: List of the avian species sampled by the authors in the Sicilian wetlands; Table S2: Spatial, hydrological, anthropic, and avian traits of the coastal wetlands of Sicily (Italy); Table S3: Description of the spatial, hydrological, and anthropic wetland traits; Table S4: Further methodological discussion.
