# Supplementary material for: Waterbird Species Are Highly Sensitive to Wetland Traits: Simulation-Based Conservation Strategies for the Birds of the Sicilian Wetlands (Italy)
Source: Biology (Basel). 2024 Apr 6;13(4):242. doi: 10.3390/biology13040242 (PMC11048633; doi:10.3390/biology13040242)
Supplement: Supplementary file 1 [file biology-13-00242-s001.zip › biology-2942010-supplementary.pdf]

Supplementary materials

Waterbird Species Are Highly Sensitive to Wetland Traits: Simulation-Based Conservation Strategies for the Birds of the Sicilian Wetlands (Italy)

Alessandro Ferrarini *, Claudio Celada and Marco Gustin

Lipu-BirdLife Italy, Via Pasubio 3, I-43122 Parma, Italy; claudio.celada@lipu.it (C.C.); marco.gustin@lipu.it (M.G.)

* Correspondence: sgtpm@libero.it; Tel.: +39-0521-1910728

Table S1. List of the avian species sampled by the authors in the Sicilian wetlands.

ID	Common name	Scientific name	Birds (2009/147/EC) Directive
1	Audouin's Gull	*Larus audouinii*	1
2	Black Stork	*Ciconia nigra*	1
3	Black Tern	*Chlidonias niger*	1
4	Black-crowned Night Heron	*Nycticorax nycticorax*	1
5	Black-headed Gull	*Larus ridibundus*	0
6	Black-necked Grebe	*Podiceps nigricollis*	0
7	Black-tailed Godwit	*Limosa limosa*	0
8	Black-winged Stilt	*Himantopus himantopus*	1
9	Broad-billed Sandpiper	*Calidris falcinellus*	0
10	Caspian Tern	*Hydroprogne caspia*	1
11	Common Greenshank	*Tringa nebularia*	0
12	Common Kingfisher	*Alcedo atthis*	1
13	Common Moorhen	*Gallinula chloropus*	0
14	Common Pochard	*Aythya ferina*	0
15	Common Redshank	*Tringa totanus*	0
16	Common Ringed Plover	*Charadrius hiaticula*	0
17	Common Sandpiper	*Actitis hypoleucos*	0
18	Common Shelduck	*Tadorna tadorna*	0
19	Common Snipe	*Galinago gallinago*	0
20	Common Tern	*Sterna hirundo*	1
21	Curlew Sandpiper	*Calidris ferruginea*	0
22	Dunlin	*Calidris alpina*	0
23	Eurasian Coot	*Fulica atra*	0
24	Eurasian Curlew	*Numenius arquata*	0
25	Eurasian Spoonbill	*Platalea leucorodia*	1
26	Eurasian Stone-curlew	*Burhinus oedicnemus*	1
27	Eurasian Teal	*Anas crecca*	0
28	Eurasian Wigeon	*Mareca penelope*	0
29	European Golden Plover	*Pluvialis apricaria*	1
30	Ferruginous Duck	*Aythya nyroca*	0
31	Gadwall	*Mareca strepera*	0
32	Garganey	*Spatula querquedula*	0
33	Great Cormorant	*Phalacrocorax carbo*	0
34	Great Crested Grebe	*Podiceps cristatus*	0
35	Great Egret	*Ardea alba*	1
36	Greater Flamingo	*Phoenicopterus roseus*	1
37	Green Sandpiper	*Tringa ochropus*	0
38	Grey Heron	*Ardea cinerea*	0
39	Grey Plover	*Pluvialis squatarola*	0
40	Gull-billed Tern	*Gelochelidon nilotica*	1
41	Kentish Plover	*Charadrius alexandrinus*	1
42	Lesser Black-backed Gull	*Larus fuscus*	0
43	Little Bittern	*Ixobrychus minutus*	1
44	Little Egret	*Egretta garzetta*	1
45	Little Grebe	*Tachybaptus ruficollis*	0
46	Little Ringed Plover	*Charadrius dubius*	0
47	Little Stint	*Calidris minuta*	0

48	Little Tern	*Sternula albifrons*	1
49	Mallard	*Anas plathyrhynchos*	0
50	Marbled Duck	*Marmaronetta angustirostris*	1
51	Marsh Sandpiper	*Tringa stagnatilis*	0
52	Mediterranean Gull	*Larus melanocephalus*	1
53	Northern Pintail	*Anas acuta*	0
54	Northern Shoveler	*Spatula clypeata*	0
55	Pied Avocet	*Recurvirostra avosetta*	1
56	Purple Heron	*Ardea purpurea*	1
57	Red knot	*Calidris canutus*	0
58	Ruddy Turnstone	*Arenaria interpres*	0
59	Ruff	*Calidris pugnax*	1
60	Sandwich Tern	*Thalasseus sandvicensis*	1
61	Slender-billed Gull	*Larus genei*	1
62	Spotted Redshank	*Tringa erythropus*	0
63	Squacco Heron	*Ardeola ralloides*	1
64	Temminck's Stint	*Calidris temminckii*	0
65	Tufted Duck	*Aythya fuligula*	0
66	Water Rail	*Rallus aquaticus*	0
67	Western Cattle Egret	*Bubulcus ibis*	0
68	Western Swamphen	*Porphyrio porphyrio*	1
69	Whimbrel	*Numenius phaeopus*	0
70	Whiskered Tern	*Chlidonias hybrida*	1
71	White Stork	*Ciconia ciconia*	1
72	Wood Sandpiper	*Tringa glareola*	1
73	Yellow-legged Gull	*Larus michahellis*	0

Table S2. Spatial, hydrological, anthropic and avian traits of the coastal wetlands of Sicily (Italy). Water salinity, diversions, and discharges were quantified on a semi-quantitative scale (0 = absent, 1 = localized, 2 = scattered, 3 = widespread), as well as tourism pressure and anthropization.

Wetland	Wetland size (ha)	Wetland isolation (m)	Distance to the coastline (m)	Mean water level (cm)	Water level fluctuations (cm)	Water salinity	Water diversions	Water discharges	Tourism pressure	Anthro-pization	Waterbird species
Biviere Gela	107.8	65,943	2,326	99.5	42.2	0	3	3	1	3	42
Gorghi Tondi	6.1	488	1,916	506.1	17.4	0	0	0	0	1	11
Laghetti Tindari	7.4	50,349	198	200.1	86.4	3	0	0	3	0	8
Lago Faro	26.9	818	409	1,849.3	1.4	2	3	2	3	3	13
Lago Ganzirri	34.1	816	990	497.6	2.6	2	3	3	3	3	17
Lago Gornalunga	13.9	63,208	0	33.9	11.9	2	0	2	2	3	28
Lago Preola	30.8	488	1,318	145.6	6.4	0	0	0	1	2	21
Pantano Auruca	31.4	461	1,009	0.0	0.0	1	3	2	0	3	1
Pantano Baronello	20.5	461	779	24.3	10.7	0	1	2	1	2	28
Pantano Bruno	16.5	1,419	1,162	11.2	6.8	0	2	2	1	3	19
Pantano Cuba	61.5	208	1,499	2.9	3.0	0	2	0	0	1	24
Pantano Grande	28.3	0	274	4.9	3.0	2	0	0	3	0	20
Pantano Longarini	109.9	208	1,674	1.2	1.5	3	3	3	3	3	8
Pantano Morghella	49.6	4,798	19	0.6	1.2	1	3	2	1	3	1
Pantano Piccolo	15.9	0	19	26.3	5.0	1	0	0	2	0	16
Pantano Roveto	124.1	628	0	14.1	10.2	3	1	2	1	1	52

Table S3. Description of the spatial, hydrological and anthropic wetland traits.

We used the GoogleEarth™ images of the region Sicily to measure wetland size (in hectares). Wetland isolation was computed as the boundary-to-boundary distance (in meters) from the nearest wetland. Distance to the coastline was the minimum distance (in meters) from the wetland boundary to the coastline. Water level (in cm) was assessed with a metric pole at each sampling point, and averaged for each date of sampling. Water level fluctuations (in cm) were calculated as standard deviation of the water levels among the dates of sampling. Salinity was evaluated indirectly through the frequency of seawater intrusions observed during field surveys. Tourism pressure measured the level of tourist activities (walking, horse riding, watersports, angling, wildfowling etc.) in the close surroundings of the wetland during field surveys. Anthropization evaluated the occurrence of greenhouses, dumpsites, quarries, camping sites, caravan parks etc. Water diversions and water discharges quantified the presence of active drainage and discharge systems.

Figure S1. A plain exemplification of how direct (E_D), indirect (E_I) and total (E_T) effects of the generic variable x_i on the alpha avian diversity (A) were calculated. In the figure below, the variable x_1 has both direct and indirect effects on A. Because the direct and indirect effects were computed from the set of partial correlation coefficients of the non-parametric Bayesian network, they range in the [-1, 1] interval.

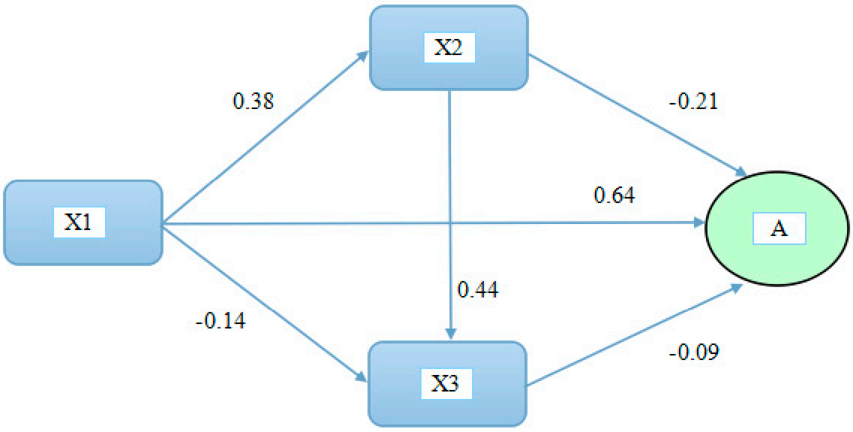

E_D = 0.64

E_I = (0.38*-0.21) + (-0.14*-0.09) + (0.38*0.44*-0.09) = -0.08

E_T = 0.64 + (-0.08) = 0.56

Figure S2. Validation of the non-parametric Bayesian network. The X axis represents the values assumed by D_N (determinant of the empirical normal rank correlation matrix; i.e. the dependence structure of the normal copula assumption) after 10^4 resampling simulations of the joint normal distribution. The Y axis shows the percentage of simulations that resulted in a certain value for D_N. The red bin indicates where D_E (determinant of the empirical rank correlation matrix; i.e. the dependence structure of the original data) fell.

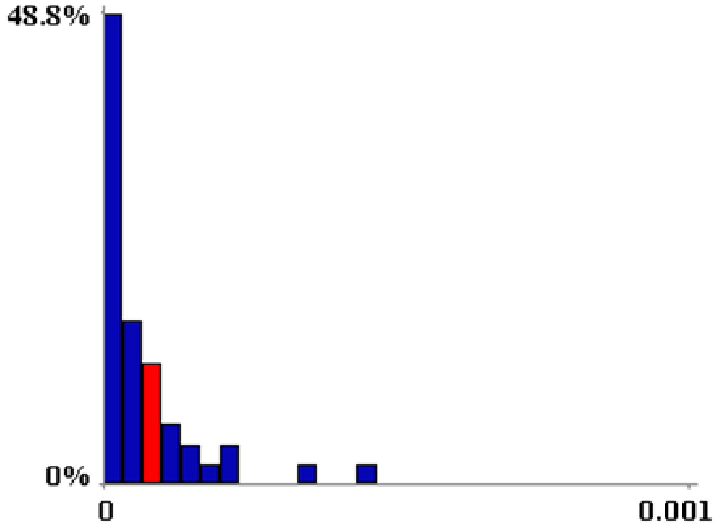

Figure S3. Alpha avian diversity (Y axis) as a function of water level fluctuations (X axis) in the Sicilian wetlands in summer (July–September). In the baseline scenario, the mean value of water level fluctuations in the studied wetlands was 13.1 cm, and the alpha avian diversity (i.e., mean number of bird species per wetland; *AD*) was 19.3. What-if simulations showed that, all other wetland traits being equal, *AD* could attain its highest value (i.e., 29.7 avian species) if water level fluctuations in all the Sicilian wetlands were equal to 30 cm, after which further increments in this variable would not produce further increase in *AD*.

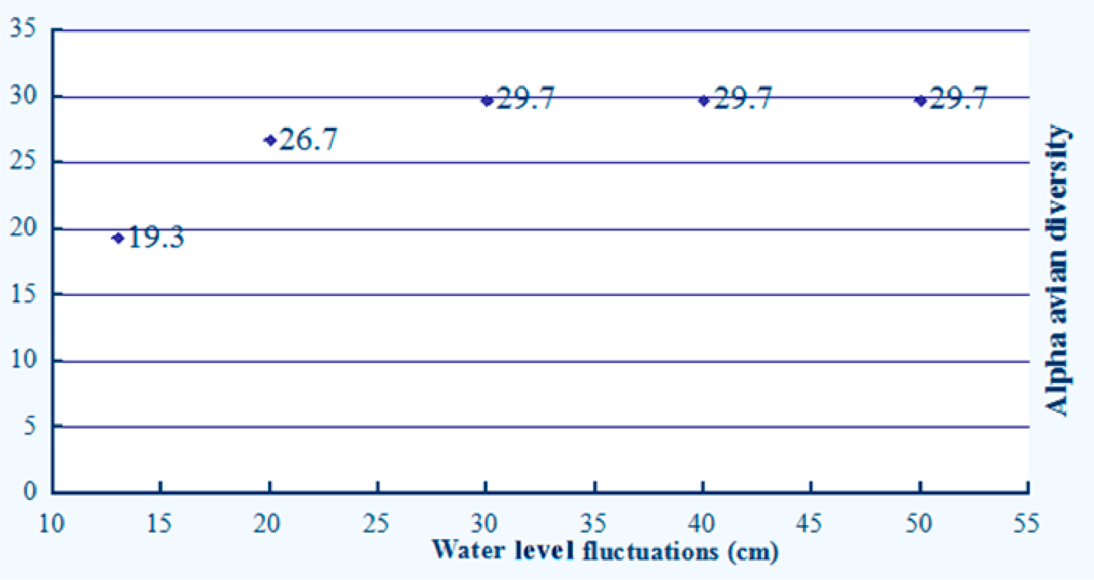

Figure S4. Map showing the geographical pattern of the alpha avian diversity in summer in the coastal wetlands of Sicily. The histogram heights (in green) are proportional to the number of bird species. The two inset maps show the level of the alpha avian diversity (the number nearby the green bar chart) for each wetland (in blue).

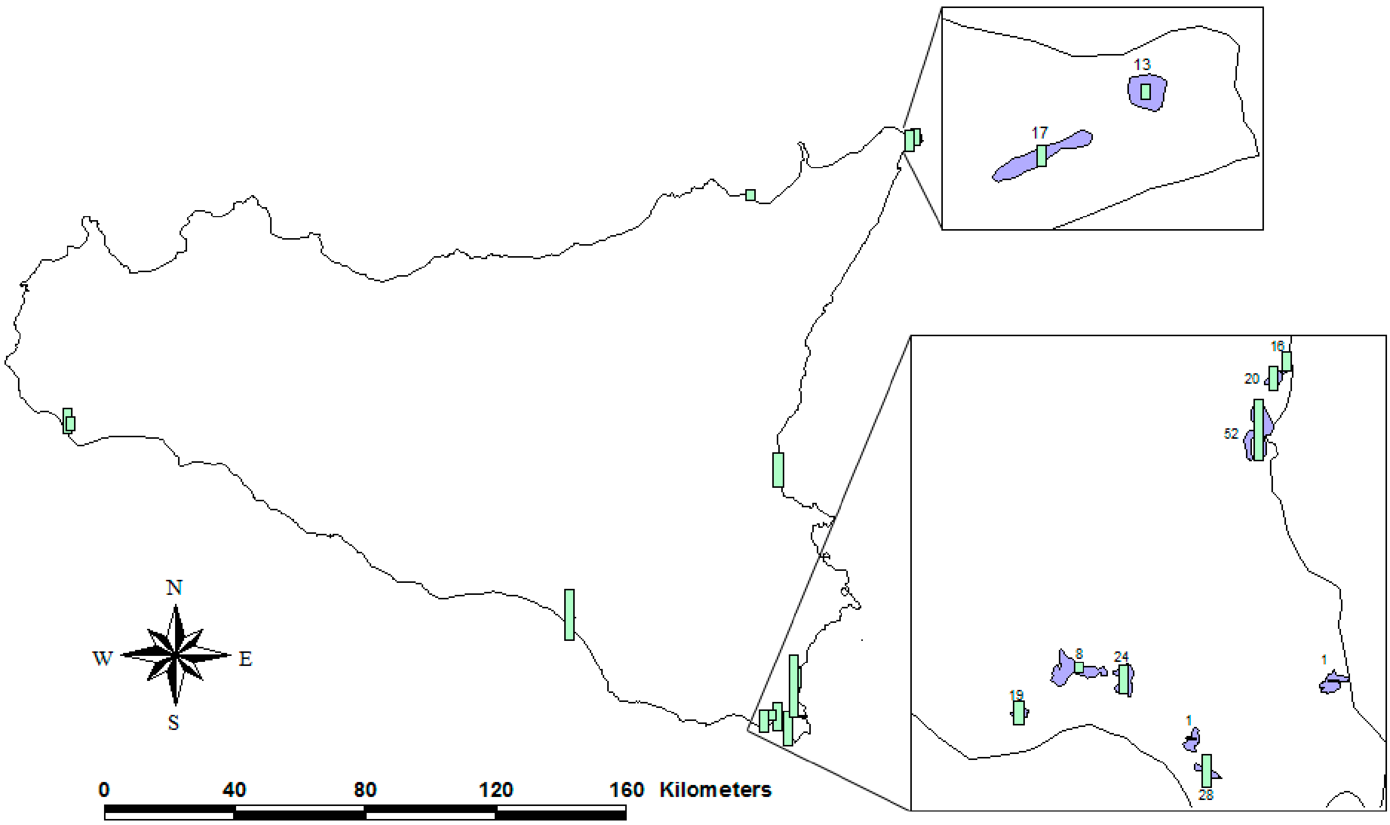

Figure S5. Conditioning of the non-parametric Bayesian network on the attributes of the wetland Pantano Bruno. The target node (i.e., avian diversity) shows the expected (i.e., most probable) number of avian species ± S.D. for such a combination of wetland traits.

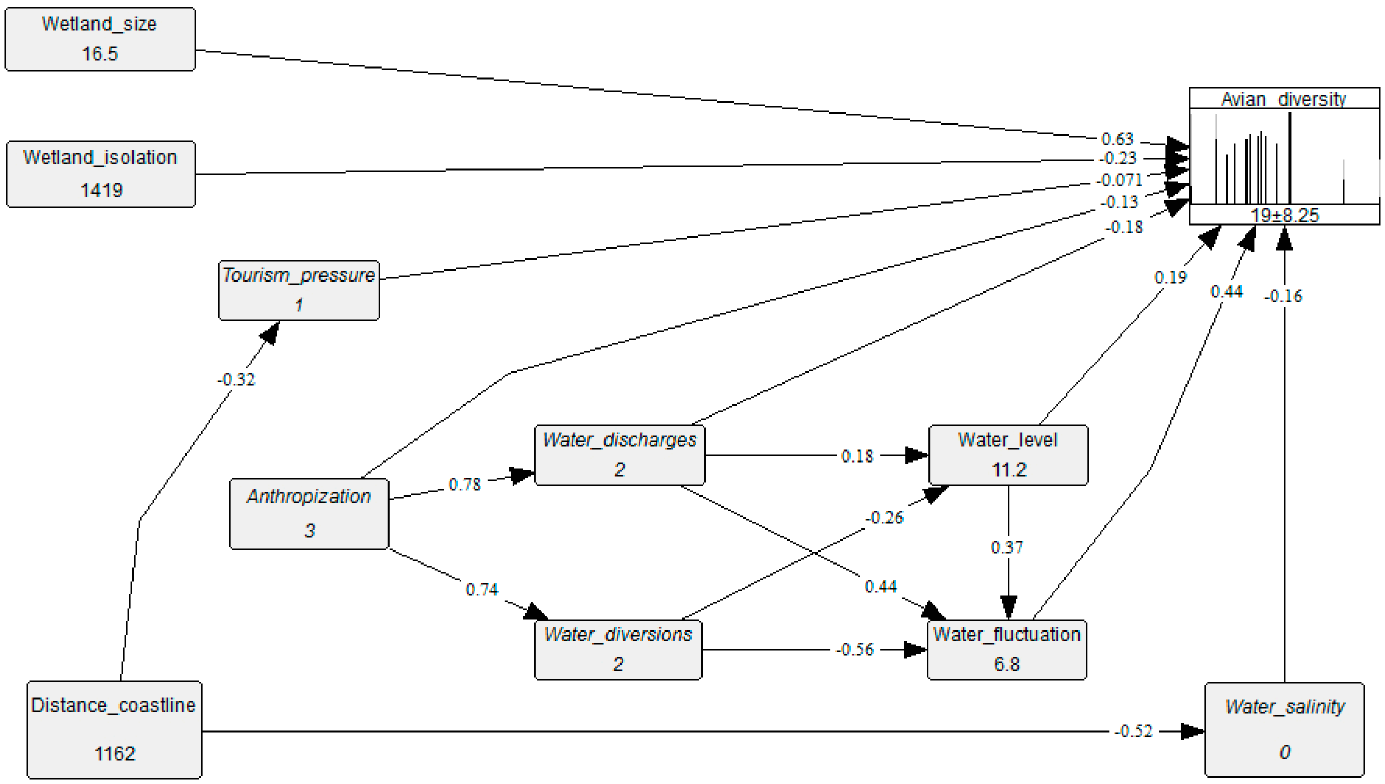

Table S4. Further methodological discussion.

In this study, we simulated the effects of changes to wetland traits upon species presences–absences. We used this kind of data because, compared to species abundances, the assessment of this ecological information is more robust during field surveys. The effects of the simulated scenarios on the alpha avian diversity could be gradual and involve, at least initially, only species abundances. However we expect that, as time goes by, the increase/decrease in abundances will at last determine the disappearance of some species and the stable occurrence of new ones, depending on whether a particular wetland (with certain spatial, anthropic and hydrological traits) can support the presence of a particular species (with certain functional traits and habitat requirements) or not.

We used three spatial variables (wetland size, isolation and distance to the coastline) as inputs to the NBN, but they were not used in the successive simulations because these wetland traits are not expected to change in the next future. However, these variables were necessary in our model because they largely influence the alpha avian diversity in the studied wetlands, and therefore allowed to precisely determine the correct partial correlations among variables, with the masking effect of the spatial traits removed. In addition, these spatial variables were used for conditioning the non-parametric Bayesian network on the values of the wetland Pantano Bruno, thus they were necessary for downscaling the NBN model locally.

Although the variable "mean water level" was present in our model, it was not directly used for successive simulations. We simulated changes to this wetland trait only indirectly, i.e. by imposing changes to the variables "*anthropization*", "*water discharges*" and "*water diversions*", which in turn increased/decreased the values of the variable "*mean water leve*l" along the pathways "*anthropization → water discharges → mean water level*" and "*anthropization → water diversions → mean water level*". The rationale is that, unlike the Sardinian wetlands where the water level in many wetlands is artificially controlled to allow for angling and fish farming, water-delivery and water-discharge systems that continuously monitor and compensate wetland hydrology are absent in Sicily; thus this type of intervention is unfeasible nowadays.

We used five proxy (ordinal) variables as inputs to our model. By definition, quantitative variables (e.g., percentage of dissolved salt in water, or cubic meters of water discharged by the surrounding facilities) are more informative than semi-quantitative ones. However, even ordinal variables could reasonably discriminate between the different levels of these variables in the studied wetlands. In addition, field surveys in all wetlands were carried out by the same experts, which assured a well-founded comparative assessment of these semi-quantitative variables.

The scenario simulations for the wetland Pantano Bruno did not involve the variable "anthropization". The presence of polytunnel greenhouses is already widespread in the close surroundings of this wetland, thus it can hardly increase. On the other hand, it is improbable that these greenhouses will be removed in the next future, therefore we handled this trait as a constant for this wetland.
